# Curious Case of Ruptured Kidney

**Published:** 1831

**Authors:** 


### LXXI.

### Curious Case of Ruptured Kidney.

A labouring man, 30 years of age, was knocked down by part of a brick-wall falling on him, and being brought to the London Hospital, appeared to have received a slight contusion on the loins. He was kept in bed, purged, and appeared to be doing very well, complaining only of weakness of the back, when, on the tenth day after the accident, he suddenly complained of severe pain in the loins. The abdomen soon afterwards became distended, great depression of the system took place, he rapidly sank, and at 3 o'clock in the afternoon he died.

On opening the abdomen there was observed behind the peritoneum a large tumour, which had pushed the viscera before it. When cut into, it appeared to consist of an enormous mass of coagulated blood, in the centre of which was the right kidney, ruptured transversely in two. The renal vessels were examined, but no trace of disease could be found, with the exception of slightly increased vascularity in a part of the lining membrane of the renal vein. The left kidney was soft, as were the two portions of the right, but they were not otherwise unhealthy.

We suppose that the kidney must have been ruptured at the time of the accident, but it is equally curious that the patient should live so long, and that he should die suddenly at last. We have heard of a case of ruptured spleen from accident, in which the patient did very well for a few days, and then died somewhat suddenly, apparently from fresh hæmorrhage. We have seen a boy live fifteen days after rupture of the liver, but then he died gradually from peritonitis occasioned by the effusion of blood and of bile into the peritoneal cavity. We regret that in the case of ruptured kidney no mention is made of the state of the urine. We never saw a case where the kidney was severely injured, in which the urine was not bloody.

---

